# Awareness, perceptions and challenges among public transport operators during the implementation of COVID-19 preventive measures in eastern Uganda: a qualitative study

**DOI:** 10.21203/rs.3.rs-3348260/v1

**Published:** 2023-10-09

**Authors:** Agnes Napyo, Leah Hopp, David Mukunya, David Soita, Joseph KB Matovu

**Affiliations:** Kabale University; The Committee on Foreign Missions of the Orthodox Presbyterian Church, Akisyon a Yesu Presbyterian Clinic; Busitema University; Busitema University; Busitema University

**Keywords:** COVID-19, public transportation, public transport operators, COVID-19 preventive measures

## Abstract

**Background:**

Public transportation plays a vital role in increasing transmission of COVID-19 due to the high-risk confined spaces in vehicles. It is therefore very crucial to employ the use of COVID-19 prevention measures during the use of public transportation to reduce risk of COVID-19 transmission. The success of the implementation and use of these measures depends largely on the public transportation users. We aimed at exploring the awareness, perceptions and challenges among public transport operators during the implementation of COVID-19 preventive measures in Eastern Uganda.

**Methods:**

This qualitative study was done in Eastern Uganda between January and February 2021. We conducted four focus group discussions, six in-depth interviews and three key informant interviews to document the awareness, perceptions and challenges faced by public transport operators including 10 boda boda riders, 19 taxi operators and 11 truck (cargo) transporters. All interviews were audio-recorded, transcribed verbatim, and analyzed using NVIVO software Version 12 plus using a thematic framework approach.

**Results:**

Generally public transport operators were aware of that COVID-19 exists, its symptoms, how it’s transmitted and ways in which it can be prevented.. However, they were not aware of what causes it and had misconceptions that it’s spread through food and mosquitoes. Meanwhile some participants perceived COVID-19 as non-existent and that it was manufactured as a biological weapon. Some COVID-19 measures were perceived as having worked well during the pandemic like putting sanctions at the country borders, vaccination, observing hand hygiene, wearing a face mask, avoiding to touch the ‘soft parts’, quarantining in a hospital setting and social distancing. The COVID-19 preventive measures perceived as having not worked well were: home isolation, covid vaccination, using alcohol-based hand sanitizer, setting up curfew time, wearing a face mask, and reducing the number of passengers in the taxis and other public transportation vehicles. Challenges faced were mainly: financial loss resulting from reduction of passengers that used public transportation and setting up of curfew time, passengers not being able to use alcohol base hand sanitizer due to religious beliefs, loss of trust in public transportation by the public, hostility and defiance from passengers, competition for passengers among public transport operators and being mistreated by implementers of COVID-19 preventive measures like police. Various key players in the implementation of COVID-19 preventive measures included: the government, health workers, media, leaders in public transport and the police.

**Conclusion and recommendation:**

Our study brings to light insights on the likely challenges that impede the use of preventive measures in public transportation use during an epidemic / pandemic like COVID-19 which could potentially escalate transmission. Focus should be put to the demystification of myths on COVID-19. Public transport passengers should be sensitized on risk of COVID-19 transmission during public transportation use and on the importance of complying with COVID-19 preventive measures. We recommend further exploration on the challenges faced by the public transportation passengers in implementing preventive measures in the event of an epidemic like COVID-19.

## Background

The coronavirus disease 2019 (COVID-19) is caused by the severe acute respiratory syndrome coronavirus 2 (SARS-CoV-2) which has affected millions of people worldwide [1]–[3]. Globally there have been over 750 million confirmed cases and about 7 million deaths as a result of COVID-19 [4]. As of January 2020, Uganda had over 170,000 confirmed cases and over 3,500 deaths as a result of COVID-19 [5]

Many governments adopted various measures to prevent the spread of COVID-19 [6]. These measures included: closure of non-essential businesses, restaurants, bars, educational institutions, preventing social gatherings, and imposing lockdowns to restrict the movement of people [7]. The Ugandan government through its Ministry of Health adopted and encouraged the massive adherence to COVID-19 preventive measures [8]. In Uganda, these measures are mainly referred to as ‘COVID-19 Standard Operating Procedures’ or ‘COVID-19 SOPs’. The terminology ‘SOPs’ has now been adopted countrywide because it was the term commonly used during the entire COVID-19 response. However, for purposes of this paper, we shall refer to them as ‘COVID-19 preventive measures’. For the case of Uganda, a number of COVID-19 preventive measures were enforced by the government and these include: getting vaccinated against COVID-19, maintaining a distance of at least 2 metres (6 feet) between persons, avoiding public gatherings and crowds, avoiding hand shaking and hugging, regularly washing hands with soap and running water or using an alcohol-based hand sanitizer, and wearing a face mask properly while always covering the nose and mouth when in public [8]. There were preventive measures that were specific for a particular context including market-places, schools, churches, work places, when using public transportation, and when conducting ‘safe’ mass gatherings [9]. In addition to the preventive measures mentioned, those that were specific for public transportation included: 1) conducting temperature screening of all individuals accessing public transportation; 2) providing hand washing facilities and ensure that these are used by all public transport users 3) reduction of the number of passengers in public transport vehicles like minibuses 4) stopping motorbikes from ferrying passengers, and 5) setting curfew time for public transportation. (i.e. all public transport vehicles had to be parked by 7:00pm) 5). Halting the use of public and private transportation vehicles from travelling from one place to the other (ie across districts)

Public transportation plays a crucial role in increasing the transmission of COVID-19 because it spreads through close contact, especially inside the confined environments in public transportation vehicles [6], [10], [11]. Uganda has a multi-modal transport system comprising of private cars, 14-seater small buses (matatus), buses, motorcycle taxis (boda bodas), cargo transportation by truck, cycling and walking. Commuters rely on informal systems dominated by matatus (46 %) and boda bodas (32 %) followed by cars (19 %), then buses (2 %) [12]. This form of public transportation is a preferred and commonly used by members of the general public because it is cheaper than using private transportation [12]. Given that public transport has been identified as a high-risk environment for transmission [11], it is important to explore user compliance to COVID-19 preventive measures for public transport operatorss during the pandemic period, to inform the potential barriers and facilitators to the implementation of preventive measures. ‘Implementation of these preventive measures is not only difficult to enforce but also very difficult to comply with. A study conducted among public transporters in Ghana found that public transport passengers hardly used any face masks[6]. Another study in Pakistan done on intentions of passengers to use preventive measures while using public transportation found that the majority of passengers never had the intention to maintain social distance, use a face mask and use a alcohol based hand sanitizer while using public transport [10]. While some public transportation operators were able to comply with some measures (including social distancing, wearing of face masks and observing hand hygiene), most public transportation systems faced challenges in ensuring that their passengers comply to using COVID-19 preventive measures [6][10]. Previous studies focused more on the period 2020 but not 2021 when the pandemic was more intense and the enforcement of prevention measures required 100% compliance to control the pandemic. It is not known, at the moment, how public transportation operators (drivers, conductors/taxi touts) managed to implement the COVID-19 preventive measures in Uganda. The purpose of this study was therefore to explore the awareness, perceptions and challenges among public transport operators during the implementation of COVID-19 preventive measures. Findings from this study will serve to inform the Ugandan government and her Ministry of Health on how to tailor context-specific preventive measures during the use of public transportation in the event of an epidemic / pandemic.

## METHODS

### Study site and setting

This study was done among public transporters in Mbale city in eastern Uganda. The public transportation sector in Uganda is categorised in to various operators that include cargo transporters, taxi (mini bus) trasnporters, boda boda (mortorcycle) riders and bus transporters. The majority of these categories of transporters have associations to which they subscribe as members. The Mbale united trucks and pick ups drivers' association is one to which the cargo transporters subscribe for their membership. The cargo transporters play a role in transporting cargo and different types of other merchandise from one place to another. They are usually long distance truck drivers. The Mbale Taxi Drivers', Conductors' and Owners' Association is subscribed to by the taxi operators. The taxi drivers normally transport passengers in 14-seater mini buses from one place to another. They are both short and long distance drivers. Taxi drivers often have individuals that assist them while they drive their passengers from place to place. These are called conductors and their role is to collect money from passengers, offloading and picking up of passengers. The Mbale motorcycle and riders association is subscribed to by the boda boda riders. Boda boda riders normally transport both cargo and passengers from place to place using motorcycles for short or long distances. However, the bus operators do not have an association for membership of bus operators. Each of these associations does have a physical office location and leadership representation which includes a chairperson, a vice chairperson, a speaker, a treasurer and so on. Each category of public transport operator will subscribe for membership to their corresponding association. The chairpersons of the associations of the various public transport operators helped in identifying the most favourable locations / venues to use during the data collection procedures. We consulted the chairpersons about the venues because they expressed concerns about the conduciveness of the venue in terms of the distance, given that they had to be closer to their business colleagues incase of any eventualities in business. The research team explained to the chairperson that the venue should qualify with the following characteristics: quiet, spacious, with good aeration, with amenities like tables, chairs, toilets etc. The in-depth interviews, key informant interviews and focus group discussions were conducted at the offices of the Mbale Taxi Drivers', Conductors' and Owners' Association. This place was selected as a venue because it was conducive given the fact that we conducted these interviews during the COVID-19 pandemic. It is an open shelter which the research team hired for the purpose of conducting the data collection. We opted for this structure because it was a well aerated, spacious and quiet environment. It was possible to observe social distancing and reduce on the possibility of COVID-19 transmission.

### Study design

This qualitative study was done between January and February 2021. We relied on the descriptive phenomenological approach in which we examined the experiences of the public transporters by interacting with them through in-depth interviews, key informant interviews and focus group discussions. The outputs of these interactions were the narrative accounts by the participants on their awareness, perceptions and challenges in the implementation of COVID 19 preventive measures while using public transportation.

### Study population and size

For purposes of this study, we only included boda boda riders, the cargo transport operators and the taxi operators because they each had a physical office location and organised leadership that made the participant identification processes more streamlined. We did not include bus operators because they do not have an association, physical office locations or organised leadership. There were forty (40) public transport operators that participated in this study including 10 boda boda riders, 19 taxi operators and 11 truck (cargo) transporters (Table 1).

### Participant selection

The public transport operators that participated in this study were purposively sampled and selected. The chairpersons at each respective association helped in identifying for the research team the various willing participants for the in depth interviews, the key informant interviews and focus group discussions. We explained the different types of data collection methods to the chairperson and the specific participant that was required for the type of data collection method. Each chairperson then gave us a list of active telephone contacts of individuals that were willing to participate. The research team then contacted the each of the willing individuals to schedule an appointment for the interview or discussion. All participants agreed to participate in the study. On meeting with each or a group of eligible individuals, the research team informed them about the study and sought consent for their participation.

### Data collection procedures and methods

We conducted focus group discussions, in-depth interviews and key informant interviews to document the awareness, perceptions and challenges faced by public transport operators. We used a key informant interview guide, in-depth interview guide, a focus group discussion guide during the interview and discussion sessions. We used three approaches to data collection to as to triangulate the data collected from the respective public transport operator category. Each of these tools consisted of open-ended, non-double barrelled questions. We requested for permission from the respondents to audio record the sessions / conversations prior to conducting our interactions. The interviews and discussions were moderated and conducted by the research team which included 2 researchers and a note taker / moderator. In the interview and discussion guides, we included guiding questions with prompts and probes to illicit more detailed responses. These guiding questions covered discussions concerning COVID-19 awareness in terms of its cause, existence, transmission, signs and symptoms, and preventive measures. We included guiding questions on the perceptions towards COVID-19 as a disease and its preventive measures as well as challenges of implementing them. Each of the interview and discussion guide tools were pilot tested to make sure that the guiding questions were clear, unambiguous and non-double barrelled. We repeated the first three interviews to clarify on responses given in the earlier interviews and included probes that had been omitted in the earlier interviews. This process of repeat interviews also helped us create rapport with the public transport operators. Field notes were taken by a moderator during the interviews and discussions to capture some unspoken observations that were not captured during the interview / discussion. Each interview lasted 45 – 60 minutes and the focus group discussions took about 60 to 90 minus long. During the interactions, the interviewers tried as much as possible to give the participants uninterrupted time / sessions while they responded during the discussions. Even if they gave an answer that did not sound right, we never castigated them for it, but continued listening to the participant. We did this in an attempt to overcome any prejudicial biases that the researchers could have had and achieve transcendental subjectivity throughout the study process. We visited each office of the association of the respective public transport operators prior to conducting the study during which time we introduced ourselves as the research team, shared the ethical approval letter and shared information about the study and the reasons for doing it.

### Data management and analysis

During the data collection phase, each interview or discussion session that was audio recorded was transcribed verbatim immediately before the next interview was conducted. The responses from each transcript were reviewed to check on any similarities and variations across different transcripts. We conducted the data collection process until we found similarities in the responses derived from the interviews and discussions. At this point we stopped collecting data and analysed the transcripts. During the review of each transcript after verbatim transcription, the text was reviewed and potential codes developed. The codes were put together to create the code book in which each code was given a description. The transcripts were imported into NVIVO software version 12 plus. Two (2) qualitative data analysts (AN and LH) organised the texts into codes using this software. Any extra codes that came up during the coding process in NVIVO were added to the code book with a corresponding description. We checked the consistency of the coding by the two (2) coders by running coding comparison query to determine the percentage of agreement and disagreement between the two (2) coders. There was a percentage agreement of 79% and a Kappa coefficient of 0.76. Each of the coders exported their codes and reference texts to Microsoft Word. The two Microsoft Word documents were reviewed by either coder and agreement on which codes to include in the results was done to avoid duplication in reporting. A single Microsoft Word document of the merged codes and reference texts was created. It is this document that we used to manually create the overall themes by categorising the existing codes. We preferred to do this manually because we were part and parcel of this text, understood the non-verbal language behind the text and context in which the text was derived. The themes derived from the codes were added to the code book. We had a meeting with the leaders of the various associations that had taken part in the study and presented to them the findings in the merged Word document. We discussed with them and made clarifications and agreements on the true representation of the findings and their experiences. At the end of the review, different codes were categorically grouped under different subthemes and a priori themes, depending on the extent to which they related with each theme. There were six a priori themes, namely:

1. Awareness on COVID-19,
2. Awareness on standard operating procedures for COVID -19 prevention,
3. Perceptions towards COVID-19 as a disease
4. Perceptions towards COVID-19 preventive measures / government directives
5. Challenges with implementing COVID -19 preventive measures
6. Minor theme: roles played by different players during the COVID-19 pandemic

We closely followed the consolidated criteria for reporting qualitative research (COREQ) during the reporting of our study findings [13].

## RESULTS

### Characteristics of public transport operators

We conducted 4 focus group discussions, 6 in-depth interviews and 3 key informant interviews involving boda boda riders, taxi operators and cargo transporters. The characteristics of the respective public transport operators are presented in table 1 and described as follows.

### Boda boda riders.

We included 10 boda boda riders in this study of whom 3 were leaders in the Mbale Motorcycle and Riders’ association, and 7 were boda boda riders. They had an average age of 39.1 years. All of them were male. 1 acted as a key informant, 2 as in-depth interviewees and 7 collectively as a focus group for the discussion. We interacted with boda boda riders between 10/2/21 and 16/2/21 (Table 2)

### Taxi Operators

We included 19 taxi operators in this study with an average age of 42.2 years. All of them were male. 3 of them were leaders of the Mbale Taxi Drivers', Conductors' and Owners' Association. 1 acted as a key informant, 2 as in-depth interviewees, 8 taxi conductors collectively as a focus group and 8 taxi drivers collectively as a focus group for the discussion. We interacted with the taxi operators between 6/2/21 and 11/2/21 (Table 2)

### Cargo transporters

We included 11 cargo transport operator in this study. All of them were male with an average age of 37.2 years.. 3 of these were leaders in the Mbale united trucks and pick-ups drivers' association. 2 responded to the indepth-interviews while 1 was a key informant and 8 took part in the focus group discussion. These interviews and discussions were conducted between 11/2/21 and 17/2/21 (Table 2)

### Awareness, perceptions and challenges among public transport operators during the implementation of COVID-19 prevention measures

The awareness, perceptions and challenges among public transport operators have been presented under the following subheadings: 1) awareness on COVID-19, 2) awareness on standard operating procedures for COVID -19 prevention, 3) perceptions towards COVID-19 as a disease, 4) perceptions towards COVID-19 preventive measures / government directives, 5) challenges with implementing COVID -19 preventive measures and 6) roles played by different players during the COVID-19 pandemic.

#### 1) Awareness on COVID 19

##### a) Awareness on what COVID 19 is

One participant mentioned that it was an unusual flu whose symptoms are more severe. However, the actual cause of COVID-19 was not known. Another participant knew that COVID-19 is caused by a virus.

***‘It is an unusual flu because with usual flu someone can sneeze and you don’t get scared but with the other type you hear like someone is sneezing from deep within. We just stop at mentioning COVID 19 but if you ask for the meaning of COVID 19, I can’t tell you, all I know is COVID 19…’*** – R13, 49 year old male.

##### b) Awareness of signs and symptoms of COVID 19

The participants were aware of various COVID 19 signs and symptoms and these included: abnormal breathing, dry cough, flu with sneezing, high temperature, joint pains, general body weakness, loss of appetite, red eyes, chest pain and headache. This is probably because of the consistent media sensitization during the COVID 19 pandemic. Some also reported that some people remain asymptomatic even with COVID-19 infections.

***If you get infected with this disease you start breathing abnormally, we don’t know what causes the abnormal breathing. The first one is abnormal breathing, the second one is having a dry cough, and the third one is having too much flu. Unusual flu because with usual flu someone can sneeze and you don’t get scared but with the other type you hear is someone is sneezing from the root of the disease’*.** – R13, 49 year old male.

***‘… then the body temperature rises... they also say that there are some people who take long to show signs.*’** – R12, 47 year old male

**‘…*development of joint pains*’** – R10, 27 year old male

***‘…he also loses appetite, those are some of the signs that I know*’** – R7, 35 year old male

**‘*They have told us that a person with COVID 19 develops red eyes…’*** – R5, 37 year old male

##### c) Awareness of how is COVID-19 is transmitted.

Some participants were knowledgeable on how COVID-19 is transmitted. One participant mentioned that COVID-19 spreads easily and quickly. Another participant expressed that given the way the taxi / mini bus is structured; it makes them sit so close to each other and puts them at risk of acquiring COVID-19. Others mentioned that COVID-19 is transmitted through air, saliva and sweat and touching contaminated surfaces. Some other participants mentioned that COVID 19 is transmitted through food and mosquitoes

***‘As taxi conductors, what we know about COVID starts with the passengers we carry, because we are always too close to each other which puts us at risk of contracting COVID.’*** – R20, 29 year old male

***‘Personally I think COVID can also be transmitted through food. If someone cooking the food is infected with COVID and his droplets fall in the food, if you eat that food I think you can also get infected, because you don’t wash the food but you just eat’.*** – R14, 25 year old male

***‘I think mosquitoes can transmit COVID because if it stings you and then it stings another person, the virus can be transmitted that way.’*** R15, 27 year old male

#### 2) Awareness on COVID 19 prevention measures.

The public transporters were very knowledgeable about COVID-19 preventive measures. There were various COVID-19 preventive measures that the public transporters reported to know about and these included: Washing hands with soap and water, wearing masks, maintaining hand hygiene using sanitizer, observing social distance, implementing nationwide lockdowns, maintaining a good diet, avoid to touch the mouth-eyes-nose area, COVID-19 testing, avoid touching contaminated surfaces, self-isolation at home, quarantine, avoiding crowded places, reducing number of passengers in the taxis, vaccination

***‘We have to wash hands with water, wear masks and also use sanitizer.’*** – R5, 37 year old male

***‘One measure is social distancing, that we should leave space between each other to protect ourselves from COVID.’*** – R9, 39 year old male

***‘We spent around four months in total lock down meaning that the Government under took a good measure to keep people in homes and it could not keep people at home for no good reason but Government had realized that the disease was serious’*** – R32, 49 year old male

***‘Personally what I know or what I tell my people, mostly drivers, my family or my friends, that they should drink a lot of lemon, drink black tea without milk and mix with lemon and eat green vegetables, there is a way these things help us to prevent COVID.’*** – R32, 49 year old male.

***‘They urge us not to touch on the mouth, nose and eyes which means that those three spots is where the virus travels very fast to enter the body. Then they should test people. That is what I would think only that Government is yet to do. What would be right is a person to first know his status as regards to COVID then after he or she starts protecting himself by using measures put in place by the ministry of health. If he / she has COVID then they do self-isolation at home.’*** – R3, 51 year old male.

***‘Not being congested in one place, staying in a well-ventilated house. Such procedures may prevent COVID 19.’*** – R30, 32 year old male.

***‘I think why they quarantine you for fourteen days is because you might have been negative at the time of testing and then you get infected the following day. So Government was right to quarantine you for the fourteen days so that they are sure that you are negative or not’.*** – R25, 44 year old male.

***‘Government told us to reduce the number of passengers in taxis. Government wanted us to practice social distancing and that’s why we were instructed to carry nine passengers and that’s what we are following.’*** – R13, 49 year old man

***‘I have heard it before over the radio stations and on televisions that COVID can be prevented through vaccination and I also heard the president saying that in a few months’ time the vaccine will be available in Uganda’.*** – R1, 48 year old male

#### 3) Perceptions towards COVID as a disease

##### a) COVID is not real / it does not exist

Even if there were participants that thought of COVID-19 as an unusual flu with serious symptoms, there were those that took COVID 19 lightly and thought that it is the normal flu and others thought that it is not even real. Other respondents actually thought that they are protected by God and that is why they didn’t suffer from COVID-19 even when they didn’t use preventive measures.

***‘Some don’t believe that COVID 19 is real. When you go to the rural areas people say that it’s a normal flu. So they have that belief and they don’t take the disease serious.’*** – R13, 49 year old male

***‘Because Ugandans have God’s mercy, we walk without any protective gear. They say we should wash our hands but some don’t wash, they say we should wear masks, others don’t wear them but it seems we are alive because of God’s mercy, but COVID is real.’*** – R8, 48 year old male

##### b) COVID 19 is not naturally occurring, it was manufactured

Some participants thought that COVID 19 is not a natural phenomenon and that it was manufactured as a biological weapon by superpower countries and was released for war to particular countries and instead just ran out of hand and spread to the rest of the world

***‘We think that the super powers are at war and one of them might have manufactured the virus as a weapon to fight other countries but it later got out of hand….’*** – R2, 47 year old male

##### c) Covid affects the elderly most

Some participants also thought that COVID 19 only affects and kills only those with a certain blood type and the elderly and that the youth are not affected as much as the elderly.

***‘That’s why you see they say that in Italy many people died of COVID but the majority of these were the elderly. But what I have seen in our country Uganda, I have not seen the youth dying a lot but it’s the older people that are dying.’*** – R32, 49 year old male

#### 4) Perceptions towards COVID 19 preventive measures / government directives

##### a) Perception towards sanctions put at the country borders by government like mandatory COVID 19 testing of truck drivers

Generally the participants thought that having mandatory testing for COVID-19 for cargo transporters at the border points as directed by the Ugandan government was so beneficial in slowing the spread of COVID-19

***‘That worked for us very well because trucks used to move to Kampala, Juba, Kenya, Rwanda, Sudan and Tanzania. So a country like Kenya had more cases of COVID than Uganda. Tanzania remained open yet they also had cases of COVID. I appreciate the Government so much for directing that one should do COVID tests before crossing borders. I remember one day a truck driver was coming from Kenya and he was stopped in Mukono after his results came out reading positive. The driver was quarantined and also treated. His truck was also sanitized. So that greatly worked for us because we remained associating freely as driver to driver.’*** – R32, 49 year old male.

##### b) Perception towards home isolation

Generally participants didn’t agree with home isolation being an effective preventive measure. Their argument leaned towards the risk that the infected person would pose to the family members and the community given the fact that our communities are social in nature. They mentioned that the government should devise means of protecting the people at home where the isolated patient would be living. The participants were more comfortable with having the COVID-19 patients isolated in a hospital setting under the care of skilled health workers because they perceived themselves as inexperienced in managing COVID-19 patients and could not afford protective gear.

***‘Personally I see that government is not helping us, because if I am positive and the doctor has proven that I am positive and then you tell me to self-isolate at home. In rural areas the roads are close to the houses and any time people pass there, the children and the old. Therefore what government is doing is not good, because they would have kept me in a safe place with doctors taking care of me.’*** – R27, 33 year old male

##### b) Perception towards covid vaccination

There were mixed perceptions towards COVID 19 vaccination. Some public transport operators were willing to be vaccinated while others did not trust the available vaccines. Those that were willing to be vaccinated, it was for the sake of saving their health and life and that of their families. The leaders were willing to vaccinate as a way of being exemplary to their staff so that their staff can also get vaccinated. Others were willing to vaccinate because it was a directive from the Ministry of Health which they trust. Other respondents trusted the vaccines because they were from World Health Organization just like the other vaccines like Hepatitis B and childhood vaccines. These vaccines have proven beneficial to the population and the COVID-19 vaccine would not be any different. Other public transport operators were sceptical about the vaccine because they had not seen other people that had been vaccinated yet. They stated that they would be willing to get vaccinated only if the doctors themselves and other people would be vaccinated first. Others were sceptical because they were unsure of the side effects. There were also those that did not believe that vaccination could prevent COVID-19 because at that time there were so many people dying of COVID-19. Some also believed that whether they are vaccinated or not, God is the one that protects them and that they can die from any other cause including the vaccination itself. Others did not trust the vaccines because they reported that there was an incidence when the Ministry of Health rejected imported COVID-19 vaccines

***‘If the vaccines arrive in Uganda when proved by the Ministry of Health that they have no harm on humans, I do accept to be vaccinated because they say prevention is better than cure. So I do go for vaccination.’*** – R6, 28 year old male

***‘By the time the vaccine is brought, it’s already confirmed. We believe that if the World Health Organization doesn’t betray us, then the vaccine can work, because all the medicine we have been using for a long time is tested first before we use it. I don’t think that they just roll out a drug to be used before testing it. Only that we don’t know the side effects of the vaccine.’*** – R2, 47 year old male

***‘I go first because I am the chairman, if I am the chairman and refuse to be vaccinated, the rest of the people may also refuse that’s why I told you in the beginning that when the ministry of health sent doctors to test truck drivers, I was the first because even when they reached police, the district police commander called me on phone, I mobilized drivers for testing in the taxi park. I told all the stage chairpersons that if someone refuses to be tested then he should be expelled.’*** – R32, 49 year old male

***‘Most people are not convinced with the vaccine, because if you had chance to hear the news yesterday, they said that there is a certain vaccine they had brought to sale to Ugandans, and the ministry rejected it because they doubted its quality. So a person who sees such news like me a low income earner may not accept to be vaccinated. At least I wash hands and wear my mask.’*** – R26, 39 year old male

***‘I don’t trust that vaccine.’*** – R23, 35 year old male

##### c) Perception towards observing hand hygiene by washing with soap and water or sanitizing with alcohol-based hand rub

One participant reported that observing hand hygiene is very helpful because it cuts the transmission of COVID-19 through the ‘soft parts’ i.e. mouth, nose and eyes. However, some had preference for using water and soap compared to alcohol-based hand sanitizer because water is readily available yet with sanitizer, there is a cost attached to buying it. One also mentioned that they preferred to use soap and water because they time it takes to wash gives them confidence that the dirt on the hands is completely washed off unlike sanitizer. There were those that preferred the sanitizer because it is convenient to carry around probably because of its convenient storage. Others were observing hand hygiene because it’s a directive by government and would be heavily fined if they didn’t follow the directive.

***‘It is helpful because it’s a way of preventing the spread of COVID through touching the face with unwashed hands. That is why they urge you to wash your hands all the time and to not touch your face so that you get used.’*** – R13, 49 year old male

***‘Water is better because it is cheaper. It is easily available and it can be used at all times. Some people can’t buy sanitizer and others can. But with water if someone comes here, he will simply wash hands there and resume with what he is doing.’*** – R40, 57 year old male

***‘As taxi conductors, it’s easier with sanitizer because you can meet a passenger in a place where there is no water and if you have your sanitizer, you can give it to him and then move on. This is better than water because you can’t move with a water container in the car but the sanitizer is easily carried and you can sanitize the passenger from anywhere you meet him. The water helps when it comes to passengers who board the taxi while at the stage or in the taxi park.’*** – R14, 25 year old male

***‘Yes both sanitizer and soap prevent COVID, but soap is better than sanitizer because with soap a person takes his time to wash properly but with sanitizer, someone comes when he is dirty, like we the taxi drivers, you stay with one bottle and you want to use it for a month, so someone comes and you spray a little in their hands then they rub without knowing what is in the hands. Therefore soap and water is better than sanitizer.’*** – R29, 51 year old male

##### d) Perceptions towards avoiding to touch the ‘soft parts’ (eyes, nose and mouth)

There are participants who thought that touching the ‘soft parts’ with dirty contaminated hands would lead to transmission of COVID-19 directly into the body. So washing hands was very important in observing this preventive measure

‘Parts like the eyes, mouth and nose easily attract the virus compared to other parts like the hand. The virus can land on your hand and stay there for the whole day without moving to another place, but the moment you touch inside your nose, the virus enters directly in your body.’ – R11, 58 year old male.

##### e) Perception towards curfew

Public transporters reported that the government setting a curfew time as a COVID-19 preventive measure during the pandemic was not helpful at all because it reduced the number of passengers that they had (especially those that would travel late in the night) and also the working hours were reduced there by impinging on their income.

***‘Curfew time has not helped us because if you have closed all sectors, what makes people move at night are the discos and bars but all these are closed, so we remain with passengers, ie he has been coming from Kampala and the taxi reaches late in town then he calls you to come pick him and take him at home. But if you just close like that, it doesn’t help. That has not prevented COVID. We who transport people have not been helped.’*** – R2, 47 year old male

##### f) Perception towards quarantine in a hospital setting.

Public transporters reported that the measure of quarantine was effective in controlling the spread of COVID-19 because it helped in having people tested and those that were found positive received treatment and prevented many COVID-19 related deaths

***‘….we managed to lock down and put people into quarantine, those that were quarantined, got chance to be treated and get cured. So we realized that the measure helped us to prevent many deaths due to COVID. So it worked.’*** – R1, 48 year old male

##### g) Perception towards wearing a face mask

One participant reported that wearing a mask works well and that it should be a mandatory measure and adhered to in the public transportation system. However, another participant mentioned that wearing a face mask was not an easy thing to do because it interferes with breathing and is not suitable for people with respiratory issues / illnesses.

***‘The measure of wearing a mask, it’s so easy and it works. If we want to fight this disease, the mask should become compulsory just like you see in other countries. If I am going to sit in a taxi, I will not sit in it if the driver is not wearing a mask and even as a driver I will not drive if the passenger is not wearing a mask. I will not sit on your bodaboda if you are not wearing a mask, and even me as the boda boda rider, I will not accept to carry a passenger who is not wearing a mask. If it’s a directive, the mask is very easy to wear.’*** – R13, 49 year old male

***‘It’s not easy to wear a mask but because of the prevailing situation, you have to wear it. Because when you wear a mask you don’t breathe well. You end up breathing in the same air that you breathe out. But it helps a bit because instead of breathing in somebody’s air, you breath in your own air. People who mostly find it hard to use the mask are those who have chronic diseases like pneumonia.*’** – R11, 58 year old male

##### h) Perception towards social distancing

Another participant reported that social distance only works if there is sufficient space between one person and another potentially infected person because one can hardly inhale the air the infected person has exhaled.

***‘It helps us in a way that if I am positive, you can’t get it because the distance helps. Even when I cough, you can’t be affected. You can’t inhale what has come out of me.’*** – R2, 47 year old male

##### i) Perception towards reducing the number of passengers in the taxis and other public transportation vehicles

Public transport operators thought that reducing the number of passengers did not work because the design of the public vehicles and motorbikes does not comply to the recommended distance for ‘social distancing’.

***‘This measure didn’t work because the width of a taxi its self is not even two meters. That didn’t work. People were congested. Distance between two people in a taxi was not even a meter so the measure didn’t work.’*** – R30, 32 year old male

#### 5. Challenges with implementing COVID-19 preventive measures

##### a) Challenge with reducing taxi passengers

The public transport operators found this measure difficult to implement because of the passengers having economic constraints, being unwilling to pay for the increased transport, and preference to sit near loved ones while using public transport. Because of all this, the transport operator were forced to let more than the recommended number of passengers into their vehicle.

***‘That directive doesn’t work because we are all not equal when it comes to income. One person can decide to sit alone, another person will prefer to share a seat with say his wife, and another will tell you that he doesn’t have money, and another will tell you that he prefers to pay half of the fare and then you are forced to add another passenger on that seat, in so doing we are not following the directives.’*** – R26, 38 year old male

***‘The problem we have been facing as transporters is that we are now loading half capacity yet some of the passengers have little money and he finds it hard to pay, yet you need more money. So you end up not agreeing with him and then you end up leaving him. But the passengers so much support the idea of adding more passengers in the taxi because of the high transport fares, and even us when we carry two passengers per seat sometimes we don’t make profit, but because you are looking for money to buy fuel, you end up accepting to take the other passenger with little money. People have also been wearing masks.’*** – R14, 25 year old male

##### b) Challenges with stopping boda boda riders from carrying any passengers

As a result of the government imposing restrictions on the number of passengers and amount of cargo to be transported via public transportation, public transporters did suffer some hardships such as being unable to buy food for their families due to the reduced income and would fail to get money from their work / business to meet extra costs.

***‘We boda boda riders don’t have any other job. You leave home when you know that you are going to ride a bodaboda. So when government stopped us from working, some of us were affected greatly because we have families, we have nowhere to get money for food, everything is needed, remember the motorbike owner needs money. He can’t let you to ride just like that. As you earn money to buy food for yourself, he also needs money. So if you get 5,000 shillings and he takes 3,000 shillings, will the 2,000 shillings be enough for you? Stopping boda bodas from operating affected us so much.’*** – R9, 39 year old male

##### c) financial handicap

The public transporters reported that they got financially handicapped because of the increased expenses related to observing COVID 19 preventive measures as directed by government. These expenses included increased tax, increased transport fares that shun away clients, increased prices for alcohol based hand sanitizer and yet their income had greatly reduced due to reduction of the number of passengers.

***‘The sanitizer is expensive, a bottle is at 8,000 shillings, the boda boda rider earns 15,000 shillings, he is supposed to give 10,000 shillings to the motorbike owner and he remains with 5,000 shillings. So what will he use to buy the sanitizer? So the issue of sanitizer on boda boda failed, the sanitizer is expensive.’*** – R7, 35 year old male

##### d) Challenge with washing hands

The pubic transporters reported that generally some passengers expressed hostility towards them when they advised them to observe hand hygiene. One reason was that sanitizer contains alcohol which is not good for the muslims. Another reason was that members of the public perceive it that they only wash hands when they are going to eat food. Public transporters also reported that it is difficult to access water and soap while in transit. So when they get passengers at places that are not major boarding stations, it is difficult to have water and soap at these transit points.

***‘But some of our people have a funny mentality, you can see an elderly person well dressed and you ask him to first wash his hands then he asks you what he is going to eat.’*** – R13, 49 year old male

***‘There are people who wash with water and they don’t like sanitizer because they say that sanitizer contains waragi (locally made alcohol), and when you tell him to sanitize, he says no and if he says no you can’t force him.’*** – R22, 47 year old male

##### e) Challenge: when political leaders don’t practice what they preach

While the government was ensuring that the public adheres to the directives on COVID-19 prevention, there were some political aspirants that went ahead to conduct public political campaigns during the pandemic without observing COVID-19 preventive measures like wearing face masks and observing social distance. This leaves the public asking themselves questions why the political leaders don’t adhere to the preventive measures yet they are the ones who are meant to be the implementers of the directives.

***‘Take an example, when we were in the political campaigns, government directed that candidates should organize meetings for only seventy people but when they realized that seventy people were few, they increased to one hundred but people would not follow this directive and would instead have meetings of two hundred people and most of these would not be wearing masks.’*** – R11, 58 year old male

##### f) Loss of trust in public transportation by the public

Due to the increased transport fares resulting from reducing the number of passengers, the members of the public lost trust in the public transportation system and its operators and as such became hostile towards the operators.

***‘Another problem we have got as taxi conductors during this COVID period is that passengers lost trust in us. Because you can tell a passenger that a journey we used to charge 4,000 we are now charging 8,000, the passenger calls you a cheat and you tell her that it’s because of COVID. Some would tell us to stop using COVID as an excuse that we are just cheats. Recently at my stage there is a passenger who cut one of the conductors with a pang because of arguing. The conductor was asking for a certain amount of money and the passenger was saying that he doesn’t have it. The passenger went and collected a pang and then cut the conductor.’*** – R21, 23 year old male

##### g) Challenges with avoiding to touch the soft parts

Some COVID-19 measures are almost impossible to implement because of natural reflexes like one’s face getting itchy while one is in a place where there is not water and soap and one ends up scratching the ‘soft parts’ (eyes, nose and mouth) without washing hands.

***‘That is unavoidable. I find myself scratching the area. I might get itchy say in the eye while in a place where there is no water, so I have to scratch myself.’*** – R13, 49 year old male

##### h) Preventive measures were not a priority for the traffic police

Public transporters reported that some key public transport law enforcers prioritized the observation of traffic rules by public transport operators over the implementation COVID-19 preventive measures. This compromised the observing of these measures by public transport operators

***‘Another thing is the traffic section of police, they have check points but a police man can come to a vehicle and not ask why someone is not wearing a mask. His interest is with only the driving permit.*’** – R13, 49 year old male

##### i) Challenges with stubborn passengers

Public transport operators reported that the passengers themselves that they carry can also be contribute to not observing of the COVID-19 preventive measures like not observing social distance in a taxi because they are in a hurry to board the vehicle that is fully loaded, not washing hands due to low risk perceptions, refusing to wear a face mask with the excuse of being unwell and so on

***‘There are vehicles that carry eighteen people, others fourteen because some passengers are stubborn, he can find passengers seated in a vehicle that is ready to move and then he says that he wants to board a taxi that is moving immediately, so he tells the other passengers to push up so that he can move quickly and when fellow passengers complain, he asks them whether it is CORONA that killed their relatives. He forces the fellow passenger to push up and then they go quarrelling for several miles.’*** – R13, 49 year old male

***‘…but you can ask a passenger to wash hands and wear a mask, then he tells you that you people are the ones who are encouraging COVID. COVID is not real people are just deceiving us.’*** – R18, 39 year old male

***‘So the challenge we have with passengers is that when you are setting off, a person accepts to wear a mask, when you look in to the driving mirror, you see when the passengers have all removed their masks. When you remind them to wear their masks, they do it but when you look away, they remove them. So I ask myself why the passengers don’t take the issue serious yet they are the ones who give us some money. Therefore, we still have that challenge with passengers. Some take it as a serious matter; others take it as a joking matter.’*** – R27, 33 year old male

##### j) Religious practices / beliefs

Some religious beliefs also hindered some passengers using public transport from using alcohol based hand sanitizer for hand hygiene. This was reported for Muslim passengers.

***‘….but for a passenger picked on the way some are even Muslims and he can tell you that sanitizer smells like alcohol so I don’t want, because you can’t move with a water container in the taxi.’*** – R19, 45 year old male

##### k) Challenge of competing for public transport users / passengers

Public transport operators reported that there was a lot of competition for passengers among public transport operators yet the number of passengers had been reduced. Because of this competition, it was hard for either the passenger and public transport operators to observe COVID-19 preventive measures like wearing face masks and hand washing. Everyone was always in a hurry to move. If one delays because of negotiating the use of COVID-19 preventive measures, then the passenger would be taken by another transport operator. According to the boda boda riders, the nature of their kind of public transportation is fast paced therefore some boda boda men find it hard to remember to implement COVID 19 preventive measures.

***‘Remember every taxi is looking for a passenger, by the time you identify a passenger and give him water to wash hands, the other passengers are being taken by your competitors because usually there are many taxis competing for passengers. So it becomes hard for a passenger picked on the way to wash hands.’*** – R19, 45 year old male

##### l) Challenge with dealing with emergence situations on the road with public transportation

For cargo transporters, the government had directed them to stop travelling with assistants as a COVID-19 preventive measure. However, they reported facing some emergency situations like car tyre punctures during the transportation of cargo on highways and having no assistance. This compromised their work in the form of delays in delivery.

***‘Take an example of a Fuso truck that is carrying ten to twelve tonnes of cargo, in case it gets a tyre puncture, as a driver you have driven for a long time and you are tired so you can’t change that tyre alone because the government stopped us from travelling with the ‘turn boy’ (assistant). It really becomes hard.’*** – R32, 49 year old male

##### m) Challenge of overcrowding at testing facilities

Cargo transport operators were subjected to mandatory testing especially at the country boarders. While at the border, the testing points did not have provision for social distancing meaning that there was overcrowding and this promoted COVID 19 spread.

***‘We get challenges when at the immigration centre because when they are testing us, they do it when we are so close to each other yet they say that we should keep social distance. I might be negative yet the other one is positive so I get worried. That is the challenge we get as truck drivers.’*** – R31, 29 year old male

### Category: mistreatment from implementers of COVID-19 preventive measures especially police.

Public transport operators generally faced brutality from the police officers when trying to implement COVID 19 preventive measures. The government had directed that 5.00pm be curfew time beyond which no public transportation modality should operate. Any public transport operator that was found operating beyond this time was charged in the courts of law or given a fine. The police was responsible for effecting this preventive measure. Public transporters reported that the police was hostile and brutal towards them when implementing the directive on curfew time.

***‘The kind of transport that we the boda boda riders have…. to the villages where we travel, they are distant and you can go and come back when it’s late. The police officers used to cane us because motor bikes had to stop moving at 5.00pm. Imagine you have gone so far at 2.00pm and you have to come back to town, when curfew time reaches before you reach home, the police officers get you and start beating you, they also ask for a lot of money yet we are not working. That is the challenge we got as bodaboda riders.’*** – R9, 39 year old male

#### 6) Roles played by different players during the COVID-19 pandemic

##### a) Role of government

The Ugandan government through her ministry of health played a role in mass sensitization of the public on COVID 19 transmission and prevention, constantly updating the public on the progress and spread of the epidemic country wide and making sure that COVID-19 measures are adhered to by the public as government directives. The public has also gained trust in the information that the government shares with them.

***‘Government has helped so much because the sensitization they did was enough and in time. If they had not sensitized us, many people would be dead by now. So Government did a great job.’*** – R12, 47 year old male

##### b) Role of health workers

The health workers play a big role in sensitizing the public and reinforcing COVID-19 preventive measures.

***‘We have to continue following guidelines given to us by the doctors.’*** – R6, 28 year old male

##### c) Role of media during the COVID-19 pandemic

Constant airing of information through many forms of media like radio, television as well as social media kept the public updated on events concerning the epidemic. Through media those that had been affected by COVID-19 were able to share their experience and in a way offered psychosocial support to members of the public

***‘Through the trainings on television and pictures that are posted on people’s phones, and some people share their experience. Government has done a lot by airing messages on radios and televisions to teach about COVID. That is why some of us are still alive and I encourage people to protect themselves.’*** – R8, 48 year old male

##### d) Role of public transport leaders

Public transport leaders played a big role in availing the materials required for use to their transport operators during the pandemic. These included books for registration (to aid contact tracing), water storage containers, soap, sanitizer, food items, etc. These leaders also play roles in sensitizing their fellow transport operators, acting as pioneers in COVID-19 vaccination so that their colleagues gain trust in it,

***‘When they asked us the managers of transport how we do it, we said that we use a book where we record a name, destination and phone contact to help us trace that person in case they give us a call asking for a certain person.’*** – R13, 49 year old male

***‘I would like to thank the chairman and the office for doing a wonderful job of sensitizing the drivers. It’s very important that the chairman has not neglected the drivers, because he has tried to sensitize them on the regulations concerning COVID. He has given us sanitizer, soap, water cans to use.*’** – R34, 45 year old male

##### e) Role of the public transporters in general

Public transport operators in general played roles in making sure that passengers followed the COVID-19 measures while using public transportation. This role started with the public transporter himself by observing the preventive measure and then making sure that the passengers followed his example.

***‘Most of the time I am the one with passengers at the back and I have to sanitize every person who enters the vehicle because he can’t leave the steering wheel to come and sanitize passengers, I have to open the door and sanitize the passenger, therefore I have to know how much sanitizer is remaining and how much soap is remaining. If any of these is finished, the driver might not know, it’s me to buy if anything is finished. If I buy, I let him know that I have bought say sanitizer. So it’s my responsibility to know what is almost running out because if I keep quiet, he might not know.’*** – R20, 29 year old male

##### f) Role of traffic police

The traffic police also played a big role in making sure preventive measures are observed during the use of public transportation by both public transport operators and users (passengers)

***‘In fact, when you meet a traffic officer, he stops you and asks you whether you have sanitized the passengers and weather they are wearing masks. If someone doesn’t have a mask, he is told to get off the taxi at that spot. Because three people were told to get off the taxi I was driving at Lugazi and we left them there. I tried to plead for them but the traffic officer threatened to give me a fine. So in order to avoid the fine, I had to leave the passengers behind.’*** – R27, 33 year old male

## Discussion

Generally public transport operators were aware of that COVID-19 exists, its symptoms, how it’s transmitted and ways in which it can be prevented.. However, they were not aware of what causes it and had misconceptions that it’s spread through food and mosquitoes. Meanwhile some participants perceived COVID-19 as non-existent and that it was manufactured as a biological weapon. Some COVID-19 measures were perceived as having worked well during the pandemic like putting sanctions at the country borders, vaccination, observing hand hygiene, wearing a face mask, avoiding to touch the ‘soft parts’, quarantining in a hospital setting and social distancing. The COVID-19 preventive measures perceived as having not worked well were: home isolation, covid vaccination, using alcohol-based hand sanitizer, setting up curfew time, wearing a face mask, and reducing the number of passengers in the taxis and other public transportation vehicles. Challenges faced were mainly: financial loss resulting from reduction of passengers that used public transportation and setting up of curfew time, passengers not being able to use alcohol base hand sanitizer due to religious beliefs, loss of trust in public transportation by the public, hostility and defiance from passengers, competition for passengers among public transport operators and being mistreated by implementers of COVID-19 preventive measures like police. Various key players in the implementation of COVID-19 preventive measures included: the government, health workers, media, leaders in public transport and the police.

### Awareness

In our study when we assessed awareness in terms of what causes COVID-19, we found that there were public transporters that did not know what caused COVID-19 while others knew that it was caused by a virus. However, all transporters were aware of the existence of COVID-19 largely because they have heard about it through the media or known someone that had been infected with it. Generally they were aware of the symptoms of COVID-19 and these have been documented in literature [2]. They were aware that COVID-19 is fatal irrespective of race, ethnic origin or class. Public transport operators were aware that COVID-19 is highly contagious and the various routes of its transmission. They were also aware that public transportation poses risk of COVID-19 transmission because of the way it is structured ie does not allow for adequate social distancing. This high risk of COVID-19 transmission via public transportation has been reported in past studies [14]. Some public transport operators thought that COVID-19 is transmitted through food and via mosquito bites which is a misconception / misinformation. The public transporters were very knowledgeable about COVID-19 preventive measures which have also been documented [2]. One study that was done to assess awareness on COVID-19 demonstrated high scores for awareness on what causes COVID-19 and its preventive measures [15]. However, the respondents in this study were health workers in Nepal. This could be explained by the frequent exposure to different forms of media.

Media in the form of radio, television, played a big role in the sensitization of the public during the COVID-19 pandemic and was the platform reported to be used by the government and ministry of health. Evidence from elsewhere demonstrates that actually the public relies on media for constant update and news during the COVID-19 pandemic and for psychosocial support for mental health purposes[16]–[19]

### Perceptions

We had public transporters that actually perceived COVID-19 as not being real and that it was just the normal flu from which the people were suffering. Others mentioned that because they were being protected by God, they had not been infected with COVID-19 even when they did not use preventive measures. Similar perceptions have been reported in the general public in Pakistan [19]. Some public transporters thought that COVID-19 was manufactured as a biological weapon by super power countries to be used for war. We also had those that thought that COVID-19 infection is selective to only those of a particular blood type and the elderly. All these perceptions are unfounded misconceptions which hinder the use of COVID-19 preventive measures and past research studies do confirm this [10], [16], [19], [20]

There were preventive measures that the public transporters perceived as effective and feasible for COVID-19 prevention these included mandatory testing at Ugandan borders, observing hand hygiene by washing with soap and water or alcohol-based sanitizer, quarantining in a hospital setting, wearing a face mask, social distancing and COVID-19 vaccination. The preventive measures that they were sceptical about included: home isolation, COVID-19 vaccination, setting in place curfew time, wearing a face mask and reducing the number of passengers in public transport vehicles. One study done in India to check the impact of the COVID-19 pandemic on travel behaviour found that public transport users believed that face masks, sanitization and vaccination were effective in preventing COVID-19 [17]. Evidence elsewhere shows that the beliefs a population has towards the preventive methods largely influence if these preventive measures will be adopted for use [21], [22].

Regarding observing hand hygiene, some public transport operators preferred alcohol-based hand sanitizer because it was convenient to carry around while others preferred water and soap because they thought it works better than alcohol-based hand sanitizer given that one takes a longer time while washing their hands. Some respondents mentioned that wearing a face mask was not an easy thing to do because it interferes with breathing and is not suitable for people with respiratory issues / illnesses.

Respondents were more comfortable with having the COVID-19 patient being quarantined in a hospital setting under the care of skilled health workers because they perceived themselves as inexperienced in managing COVID 19 patients and not being able to afford protective gear. However, several respondents didn’t agree with home isolation being an effective preventive measure. Their argument was that infected person would pose a risk for infection to the family members and the community given the fact that our communities are social in nature.

A number of studies also shown that these preventive measures cause some undesired social effects with in the population eg social distancing caused [20], [23]

There were mixed perceptions towards COVID 19 vaccination. Some public transport operators were willing to be vaccinated for the sake of saving their life and that of their families. The leaders were willing to vaccinate as a way of being exemplary to their staff so that their staff can also get vaccinated. Others were willing to vaccinate because it was a directive from the ministry of health which they trust. Other respondents trusted the vaccines because they were from the World Health Organisation just like the other vaccines like Hepatitis B and childhood vaccines which have proven beneficial to the population and the COVID 19 vaccine would not be any different. Other public transport operators were sceptical about the COVID-19 vaccine because they did not trust them since they had not seen other people that had been vaccinated at that time. They stated that they would be willing to get vaccinated only if the doctors themselves and other people would be vaccinated first. Others were sceptical because they were unsure of the side effects.

Public transporters reported that the government setting a curfew time as a COVID-19 preventive measure during the pandemic was not helpful at all because it reduced the number of passengers that they had and yet it is these passengers that pay for their service with the money that supports their livelihood.

Public transport operators thought that reducing the number of passengers was not ideal because the design of the public vehicles and motorbikes does not comply to the recommended distance for ‘social distancing’. In other settings, a reduction in the use of public transportation was seen [10], [17], [24] probably because the passengers were afraid of contracting COVID-19 infection given that the structure of the public transportation vehicles does not comply with the recommended social distancing gap between people. The risk of COVID-19 transmission in public transportation has been demonstrated in China [11], [14].

One of the challenges faced by public transporters during the implementation of the COVID-19 preventive measures was being financially handicapped. This financial handicap was as a result of the reduction of the number of passengers to half in taxis which led to increased public transport fares and reluctance of the public to use public transportation. The boda boda riders were also stopped from carrying passengers. For all public transport operators, curfew time was put in place which drastically reduced their working hours. The resulting financial handicap resulted in public transport operators being unable to fend and take care of their families. Our findings are similar to evidence found elsewhere [19], [25] that various forms of public transport suffered reduction in numbers of users and thus suffering economic loss. However, one way that this was brought thought to be reduced was to have the public transporters cost share the high transport costs with the government and users of public transport [25]

The passengers themselves also became a challenge in the use of public transportation. Some passengers were hostile towards the public transport operators especially when advised to observe COVID-19 measures like washing hands and wearing of face masks. A study done in Ghana also found that passengers in public transport didn’t comply to wearing of face masks [6]. Studies done elsewhere in Uganda [26]have demonstrated reluctance in social distancing [27]. Passengers also lost trust in the public transport operators because of the increased public transport fares. However, the very study done in Ghana showed that public transport users were willing to cost share in the prices attached to the procurement of preventive measure items in public transportation by paying a higher transport fare [25] probably because they had better sensitization.

Public transport operators reported that there was a lot of competition for passengers among public transport operators yet the number of passengers had been reduced. Because of this competition, it was hard for either the passenger and public transport operators to observe COVID 19 preventive measures like wearing face masks and hand washing.

For cargo transporters, the government had directed them to stop travelling with assistants as a COVID 19 preventive measure. However, they reported facing some emergency situations like car tyre punctures on during the transportation of cargo on highways and having no assistance. This compromised their work in the form of delays in delivery.

Cargo transport operators were subjected to mandatory testing especially at the country boarders. While at the border, the testing points did not have provision for social distancing meaning that there was overcrowding and this promoted COVID 19 spread.

Public transport operators generally faced brutality from the police officers when trying to implement COVID 19 preventive measures. The government had directed that 5.00pm be curfew time beyond which no public transportation modality should operate. Any public transport operator that was found operating beyond this time was charged in the courts of law or given a fine. The police was responsible for effecting this preventive measure. Public transporters reported that the police was hostile and brutal towards them when implementing the directive on curfew time,

Accessing water and soap during transit times, yet sanitizer is expensive for public transporters. It was also difficult for Muslim passengers to use alcohol-based hand sanitizer as this is a taboo in their religion.

Avoiding touching the eyes, nose and mouth was reported to be a challenge because of the interference of response to natural reflexes like scratching oneself when the eye or nose is itching.

Public transport law enforcers (traffic police) prioritized the observation of traffic rules by public transport operators over the implementation COVID 19 preventive measures. This compromised the observing of these measures by public transport operators

Generally every member of the public has a role to play in public transportation during an epidemic. This includes the government and its ministry of health, health workers, the public transport leaders and operators, the public transport users, the traffic police. These roles include: Sensitization. Trusting the process, Reinforcing covid-19 prevention measures, Availing to the public what is required for prevention

## Conclusions and recommendation

Our study brings to light insights on the likely challenges that impede the use of preventive measures in public transportation use during an epidemic / pandemic like COVID-19 which could potentially escalate transmission. Focus should be put to the demystification of myths on COVID-19. Public transport passengers should be sensitized on risk of COVID-19 transmission during public transportation use and on the importance of complying with COVID-19 preventive measures. We recommend further exploration on the challenges faced by the public transportation passengers in implementing preventive measures in the event of an epidemic like COVID-19.

### Limitations

We never included the views and experiences of the public transport user and other key players in public transportation like the traffic police officers.

## Figures and Tables

**Table 1:** showing number and distribution of study participants by data collection method

Data collection method	Category and number of participants		
	Bodaboda riders	Taxi operators	Cargo transporters
FGDs	1 FGD (with 7 boda boda riders)	2 FGDs (1 FGD consisted of 8 taxi drivers and the 2^nd^ FGD consisted of 8 taxi conductors)	1 FGD (with 8 truck drivers)
In-depth interviews	2 (with 2 leaders of the association of boda boda riders)	2 (with 2 of the leaders of the association of taxi operators)	2 (with 2 of the leaders of the association of truck drivers)
Key informant interviews	1 (with 1 leader of the association of boda boda riders)	1 (with 1 leader of the association of taxi operators)	1 (with 1 leader of the association of taxi operators)
**Total No. of participants per category of public transport operator**	**10**	**19**	**11**
**Total number of participants**	**40**		

**Table 2:** Characteristics of public transport operators in Eastern Uganda.

ID No	Age in years	Role of public transport operator	Gender	Type of interview / discussion
Boda boda riders / operators				
R1	48	Assistant speaker of boda boda association	Male	Key informant interview
R2	47	Speaker	Male	In-depth interview
R3	51	Coordinator	Male	In-depth interview
R4	31	Boda boda rider	Male	Focus group discussion
R5	37	Boda boda rider	Male	Focus group discussion
R6	28	Boda boda rider	Male	Focus group discussion
R7	35	boda boda rider	Male	Focus group discussion
R8	48	Boda boda rider	Male	Focus group discussion
R9	39	Boda boda rider	Male	Focus group discussion
R10	27	Boda boda rider	Male	Focus group discussion
Taxi drivers / operators				
R11	58	Vice Chairperson of taxi association	Male	Key informant interview
R12	47	Chairperson of taxi association	Male	In-depth interview
R13	49	Chairperson of road station	Male	In-depth interview
R14	25	Taxi conductor	Male	Focus group discussion
R15	27	Taxi conductor	Male	Focus group discussion
R16	23	Taxi conductor	Male	Focus group discussion
R17	25	Taxi conductor	Male	Focus group discussion
R18	39	Taxi conductor	Male	Focus group discussion
R19	45	Taxi conductor	Male	Focus group discussion
R20	29	Taxi conductor	Male	Focus group discussion
R21	23	Taxi conductor	Male	Focus group discussion
R22	47	Taxi driver	Male	Focus group discussion
R23	35	Taxi driver	Male	Focus group discussion
R24	43	Taxi driver	Male	Focus group discussion
R25	44	Taxi driver	Male	Focus group discussion
R26	39	Taxi driver	Male	Focus group discussion
R27	33	Taxi driver	Male	Focus group discussion
R28	44	Taxi driver	Male	Focus group discussion
R29	51	Taxi driver	Male	Focus group discussion
Cargo transport operators (truck drivers)				
R30	32	Speaker of association	Male	In-depth interview
R31	29	Treasurer of association	Male	In-depth interview
R32	49	Chairperson of association	Male	Key informant interview
R33	23	Truck driver	Male	Focus group discussion
R34	45	Truck driver	Male	Focus group discussion
R35	37	Truck driver	Male	Focus group discussion
R36	33	Truck driver	Male	Focus group discussion
R37	29	Truck driver	Male	Focus group discussion
R38	42	Truck driver	Male	Focus group discussion
R39	33	Truck driver	Male	Focus group discussion
R40	57	Truck driver	Male	Focus group discussion
